# Development of a novel vitrification method for chondrocyte sheets

**DOI:** 10.1186/1472-6750-13-58

**Published:** 2013-07-25

**Authors:** Miki Maehara, Masato Sato, Masahito Watanabe, Hitomi Matsunari, Mami Kokubo, Takahiro Kanai, Michio Sato, Kazuaki Matsumura, Suong-Hyu Hyon, Munetaka Yokoyama, Joji Mochida, Hiroshi Nagashima

**Affiliations:** 1 Laboratory of Developmental Engineering, School of Agriculture, Meiji University, 1-1-1 Higashimita, Tama, Kawasaki, Japan; 2 Meiji University International Institute for Bio-Resource Research (MUIIBR), 1-1-1 Higashimita, Tama, Kawasaki, Japan; 3 Department of Orthopaedic Surgery, Surgical Science, Tokai University School of Medicine, 143 Shimokasuya, Isehara, Kanagawa, Japan; 4 Laboratory of Microbial Genetics, School of Agriculture, Meiji University, 1-1-1 Higashimita, Tama, Kawasaki, Japan; 5 School of Materials Science, Japan Advanced Institute of Science and Technology, 1-1 Asahidai, Nomi, Ishikawa, Japan; 6 Center for Fiber and Textile Science, Kyoto Institute of Technology, Creation Core Kyoto Mikuruma 213, Kamigyo, Kyoto, Japan

**Keywords:** Cell sheet therapy, Chondrocyte sheet, Vitrification, Cryopreservation, Cartilage repair

## Abstract

**Background:**

There is considerable interest in using cell sheets for the treatment of various lesions as part of regenerative medicine therapy. Cell sheets can be prepared in temperature-responsive culture dishes and applied to injured tissue. For example, cartilage-derived cell sheets are currently under preclinical testing for use in treatment of knee cartilage injuries. The additional use of cryopreservation technology could increase the range and practicality of cell sheet therapies. To date, however, cryopreservation of cell sheets has proved impractical.

**Results:**

Here we have developed a novel and effective method for cryopreserving fragile chondrocyte sheets. We modified the vitrification method previously developed for cryopreservation of mammalian embryos to vitrify a cell sheet through use of a minimum volume of vitrification solution containing 20% dimethyl sulfoxide, 20% ethylene glycol, 0.5 M sucrose, and 10% carboxylated poly-L-lysine. The principal feature of our method is the coating of the cell sheet with a viscous vitrification solution containing permeable and non-permeable cryoprotectants prior to vitrification in liquid nitrogen vapor. This method prevented fracturing of the fragile cell sheet even after vitrification and rewarming. Both the macro- and microstructures of the vitrified cell sheets were maintained without damage or loss of major components. Cell survival in the vitrified sheets was comparable to that in non-vitrified samples.

**Conclusions:**

We have shown here that it is feasible to vitrify chondrocyte cell sheets and that these sheets retain their normal characteristics upon thawing. The availability of a practical cryopreservation method should make a significant contribution to the effectiveness and range of applications of cell sheet therapy.

## Background

The use of cell sheets is being actively investigated in the field of regenerative medicine as a potential treatment for various lesions [1,2]. For example, Okano et al. developed a method of preparing various types of cell sheets using temperature-responsive culture dishes [3]; additionally, cell sheets derived from corneal epithelia [4], skin [5], oral mucosal epithelia [6], bladder epithelia [7], myocardial cells [8,9], periodontal ligaments [10], and cartilage [11,12] are currently under investigation in preclinical studies or clinical applications [13-15].

We have been investigating the use of chondrocyte-derived cell sheets for treatment of knee cartilage injuries [11,12,16-18]. Cell sheets can be used as autografts or allografts. In a clinical setting, the preparation of autologous cell sheets involves a defined period of time for culture of the cells. Thus, the timing of transplantation has to be arranged with regard to both the needs of the patient and the condition of the cultured cell sheet. Cryopreservation of cell sheets would simplify the coordination of transplantation timing and would also allow repeated treatments for a single patient. In addition, development of robust cryopreservation methods and distribution protocols would need to be established to facilitate allograft-based cell sheet therapy. There is little doubt that cryopreservation of cell sheets would provide significant benefits to clinical applications of cell sheet therapies.

Cell sheet therapy involves covering a tissue lesion with a membranous sheet [11,19,20]. Therefore, an indispensable prerequisite of a cryopreservation method is to maintain the integrity of the membranous structure of the cell sheet. However, achieving this has been challenging and although the viability of the cells comprising the sheets can be maintained, damage to the integrity of the sheet often occurs [21]. To date, no practical cryopreservation method has been developed for cell sheets that have been generated in temperature-responsive culture dishes.

Recent developments in vitrification methods have allowed practical cryopreservation of early stage embryos of many mammalian species, including humans [22,23]. Embryos are highly susceptible to damage by various aspects of cryopreservation, including the toxicity of the cryoprotectant (CPA), osmotic shock, and temperature shock [23,24], which makes their successful cryopreservation more difficult compared to somatic cells. Furthermore, embryos of some mammalian species such as pigs [25-28] are especially sensitive to low temperatures. However, the latest vitrification methods have enabled very high post-cryopreservation survival rates for mammalian embryos of a wide range of species, derivations or backgrounds [23,29,30]. The basic principle of the latest high-performance method involves vitrifying embryos with a very small amount of vitrification solution, a process termed the minimum volume cooling (MVC)-vitrification method [29-32]. The MVC-vitrification method is effective at stabilizing the vitreous status of the solution during vitrification and rewarming, and thereby achieves a high rate of survival of embryos.

In light of the success achieved in embryos, we decided to apply the basic principles of the MVC-vitrification method for cryopreservation of chondrocyte sheets. We successfully developed a coating method by which a cell sheet could be vitrified using a minimum amount of solution. This report describes the development of an effective vitrification method that does not impair either the macro- or microstructures of cell sheets, and thereby possesses significant potential for applications related to clinical cell sheet therapy.

## Methods

### Chemicals

All chemicals were purchased from Sigma-Aldrich Chemical Co. (MO, USA), unless otherwise indicated.

### Preparation of rabbit chondrocyte sheets

The study was conducted using commercially available primary cultured cells (Primary Cell, Hokkaido, Japan) derived from the knee cartilage of a Japanese white rabbit. The cells were plated onto temperature-responsive culture dishes (UpCell, diameter: 35 mm; CellSeed, Tokyo, Japan) [3] at a density of 5.0 × 10^4^ to 1.0 × 10^5^ cells/dish and cultured in RPMI-1640 medium (11875; GIBCO, Life Technologies Corporation, CA, USA) supplemented with 10% fetal bovine serum (FBS; 171012, Nichirei Biosciences, Tokyo, Japan) at 37°C in a humidified atmosphere of 5% CO_2_ in air. The medium in each dish was replaced with the same medium supplemented with 100 μM L-ascorbic acid (A4544; Wako Pure Chemical Industries, Osaka, Japan) when the cells reached confluence. The cells formed a single thin layer after 2 weeks of plating, at which time the UpCell dishes were placed at 25°C for 30 min to promote detachment of the cell sheet from the bottom surface of the dish [11]. The cell sheet was then removed from the dish using a cell shifter (CellSeed). Three cell sheets were layered together to form a triple-layered sheet, and this sheet was further cultured for 1 week in the UpCell dish.

### Vitrification solutions

The vitrification solution developed for cryopreserving mammalian embryos [22,32] was used after slight modifications.

Hepes (20 mM) buffered Tissue Culture Medium-199 (Nissui Pharmaceutical, Tokyo, Japan) supplemented with 20% calf serum (12133C; SAFC Biosciences, KS, USA) was used as the basal solution. Dimethyl sulfoxide (DMSO) and ethylene glycol (EG) were used as permeable CPAs. Sucrose was used as a non-permeable CPA for all vitrification experiments. In some experimental groups, carboxylated poly-L-lysine (COOH-PLL) [33] was also added as a supplemental non-permeable CPA.

An equilibration solution (ES) consisting of 10% (v/v) DMSO and 10% (v/v) EG in the basal solution and a vitrification solution (VS) containing 20% (v/v) DMSO, 20% (v/v) EG, and 0.5 M sucrose were prepared. The effect of supplementation of the VS with 10% (w/v) COOH-PLL was examined in some of the experimental groups. A rewarming solution (RS) and a dilution solution (DS) containing 1 M and 0.5 M sucrose, respectively, were prepared and the basal solution was used as the washing solution (WS).

The VS was used at ice temperature (on crushed ice), and pre-warmed RS at 38°C was used to devitrify (rewarm) the vitrified cell sheet. All other solutions were used at room temperature (24-27°C).

### Vitrification procedures

#### Coating method

**Figure 1:**
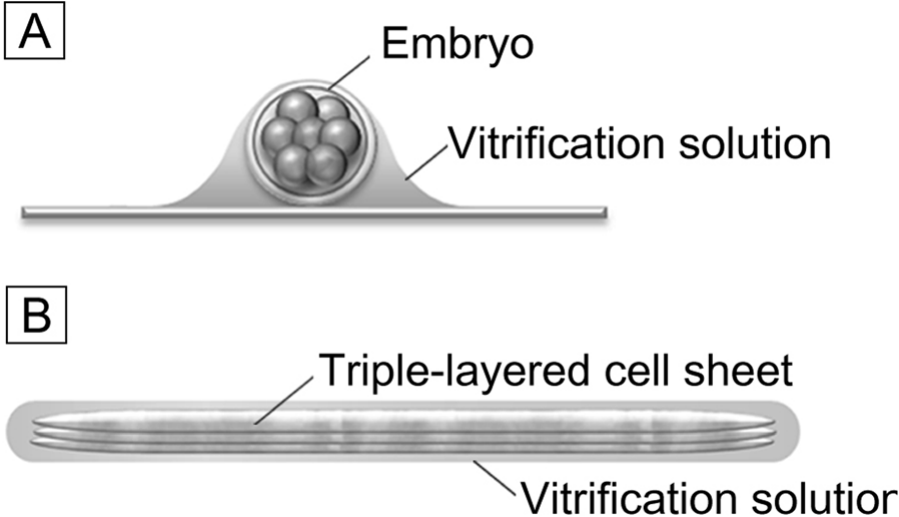
**The minimum volume cooling concept applied to vitrification of an embryo and a cell sheet. A**: The protocol of embryo vitrification using the minimum volume cooling (MVC) concept; the embryo is vitrified in a minimum volume of vitrification solution on a plastic or metal plate with high thermal conductivity [22,32]. **B**: For cryopreservation of triple-layered rabbit chondrocyte sheets, we developed a coating method in which the cultured cell sheet is thinly coated with a viscous vitrification solution containing permeable and non-permeable cryoprotectants. This enables the cell sheet to be vitrified in a minimum amount of vitrification solution in accordance with the MVC concept.

The development of the coating method for the chondrocyte sheets was guided by the MVC concept [31], which has been shown to be effective for the vitrification of mammalian embryos. The method used here involves vitrification of a cell sheet; this is achieved by treating a cell sheet with ES and VS and then applying a thin coat of VS containing permeable and non-permeable CPAs. This technique enables vitrification of the cell sheet with the minimum amount of vitrification solution (Figure 1).

**Figure 2:**
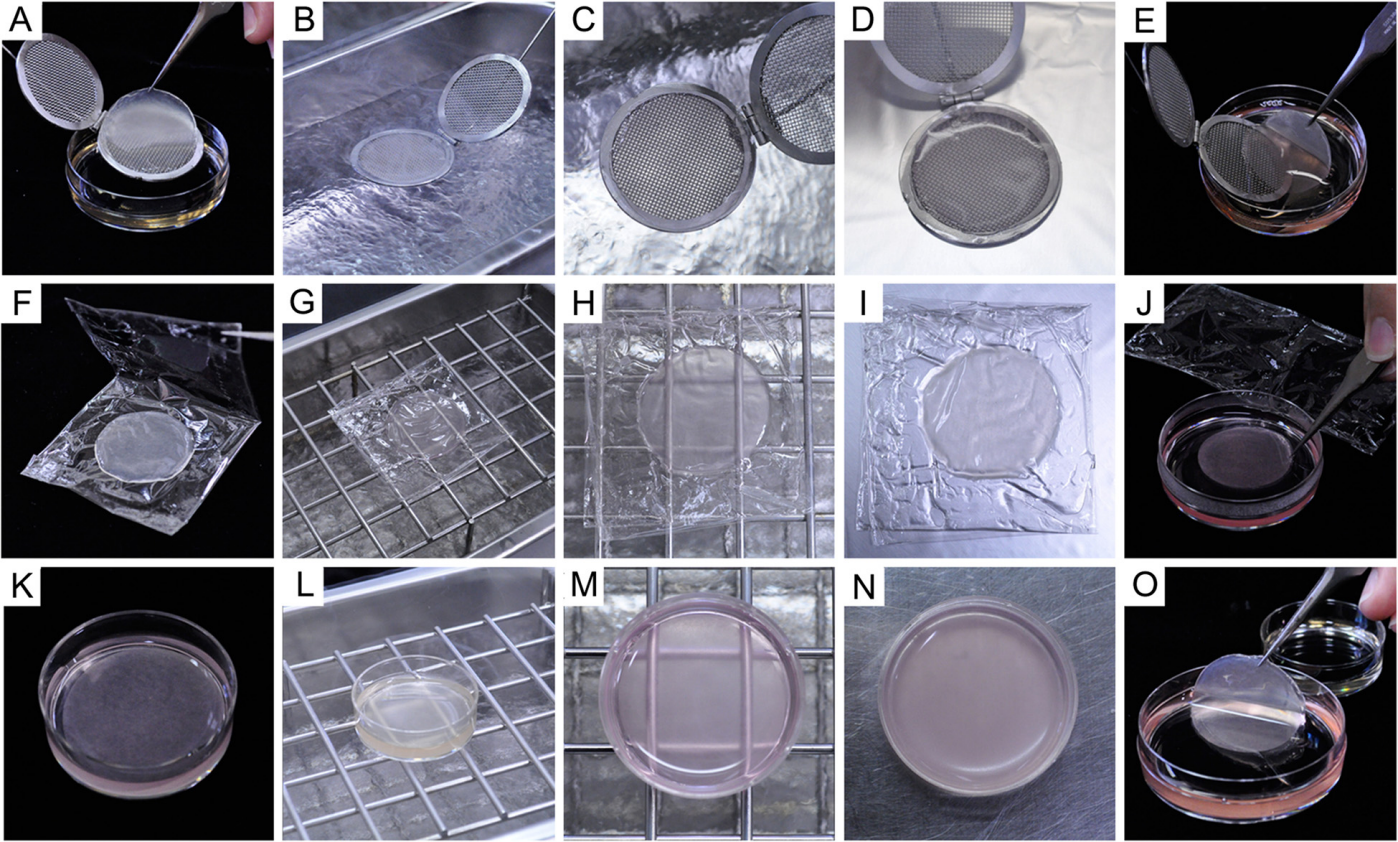
**Vitrification and rewarming methods for triple-layered rabbit chondrocyte sheets. (A**-**E)** Coating method. A cell sheet treated with ES and VS is placed on a stainless mesh using forceps **(A)** and exposed to liquid nitrogen (LN) vapor for vitrification **(B)**. **(C)** A cell sheet vitrified on mesh. Note the transparency of the vitrified sheet. For rewarming, the vitrified sheet on a mesh device is placed on a heating plate for thawing **(D)** and then transferred to RS using forceps **(E)**. **(F**-**J)** Wrapping film method. An ES- and VS-treated cell sheet was wrapped in an envelope of polyvinylidene chloride film **(F)** and exposed to LN vapor for vitrification **(G)**. **(H)** A cell sheet vitrified in wrapping film. Note that the cell sheet is transparent when it solidifies. For rewarming, the sheet vitrified in the wrapping film is placed on a heating plate **(I)**, followed by transfer into RS **(J)**. **(K**-**O)** Dish method. A cell sheet pretreated with ES and VS is placed in 2 ml VS **(K)** and exposed to LN vapor for vitrification **(L)**. **(M)** A cell sheet solidified with VS in a dish. Note the transparency of the vitrified solution and the lack of any cracks. **(N)** A dish containing a cell sheet on a heating plate for rewarming. The opacity of the solution indicates that ice crystals have formed during the warming process. The thawed cell sheet is then transferred into RS using forceps **(O)**.

First, a triple-layered cell sheet was peeled from the UpCell surface using a cell shifter and forceps, and immersed in 5 ml of ES in a 60 mm dish (Iwaki 3010–060, AGC Techno Glass, Shizuoka, Japan) for 5 min for pre-equilibration. Then, the cell sheet was transferred to the same solution in a fresh dish for 20 min (equilibration). After equilibration, the cell sheet was transferred to VS using forceps for 5 min (VS pre-treatment), and then transferred to fresh VS in another dish for 15 min for dehydration and the permeation of the permeable CPAs. VS containing COOH-PLL and unmodified VS were compared. After VS treatment, the cell sheet was carefully placed on a stainless steel mesh (30 mm) using forceps (Figure 2A). The cell sheet on the mesh was held 1 cm above the surface of liquid nitrogen (LN) in a 1 L Styrofoam bath and was vitrified by a 20 min exposure to the LN vapor (−140°C) (Figure 2B). We observed that vitrification of the cell sheet was completed within the first 5 min (Figure 2C). The use of LN vapor was adopted after preliminary tests demonstrated that the cell sheet fractured when it was directly immersed in LN.

To rewarm a vitrified sheet, the mesh holding the sheet was placed directly onto an electric heating plate (HP-4530; ASONE Corporation, Osaka, Japan) at 38°C for 90 sec (Figure 2D). After the sheet had completely thawed, the mesh holding the sheet was slowly placed into 10 ml of RS in a 60 mm dish and was gently removed from the mesh using forceps (Figure 2E). At this stage, the recovered sheet was checked for cracks. The CPAs were diluted and removed in a stepwise manner. Briefly, the cell sheet was held in RS for 1 min and then transferred into 5 ml of DS using forceps for 3 min. Then, the cell sheet was transferred twice into 5 ml of WS. The cell sheet was gently shaken several times in each solution to help diffusion of the CPAs.

#### Envelope method

A cell sheet pre-treated with ES and VS as described above in the coating method, was placed onto a 5 × 10 cm rectangular piece of polyvinylidene chloride kitchen wrap (Kureha Corporation, Tokyo, Japan) using forceps. Then, the wrapping film was folded to enclose the cell sheet (Figure 2F). The wrapped sheet was held 1 cm above the surface of LN and vitrified by exposure to the vapor for 20 min (Figure 2G). Vitrification of the cell sheet was observed within the first 5 min (Figure 2H). As the presence of COOH-PLL in the VS was found to be essential for maintaining the structure of the vitrified sheet in the development of the coating method described above, VS containing COOH-PLL was used for the envelope method.

To rewarm the vitrified sheet, the cell sheet envelope was placed directly onto a heating plate at 38°C for 90 sec (Figure 2I). When the sheet had completely thawed, the wrapping film was slowly opened and the sheet was transferred into RS using forceps (Figure 2J). The recovered sheet was checked for cracks, and the CPAs were diluted, removed, and washed as described above for the coating method.

#### Dish method

In order to evaluate the effect of VS volume on cell sheet vitrification, a sheet was immersed in 2 ml of VS, a considerably larger volume than used in the coating or envelope methods.

A preliminary study revealed the formation of many cracks and ice crystals when the vitrification/rewarming procedure was performed using 2 ml of VS in a 35 mm plastic dish (Additional file 1: Figure S1A). However, ice crystal and crack formation was significantly reduced when the VS contained 10% COOH-PLL (Additional file 1: Figure S1B). Therefore, the dish method was performed using VS containing COOH-PLL.

A cell sheet was pretreated with ES and VS in the same manner as in the coating and envelope methods, placed in 2 ml of VS in a 35 mm dish (Nunc 150318, Thermo Fisher Scientific, Kanagawa, Japan) for 15 min for further CPA permeation and dehydration (Figure 2K). The dish was held 1 cm above the surface of LN for 20 min (Figure 2L) to induce solidification of the VS containing the sheet (Figure 2M).

To rewarm the vitrified sheet, the dish was allowed to stand for 3 min on a heating plate at 38°C (Figure 2N). After the complete melting of the VS in the dish, the cell sheet was transferred into RS using forceps (Figure 2O) and checked for visible damage (cracks). The CPAs were diluted, removed, and washed in the same manner as in the coating method.

### Survival of the cells in the vitrified cell sheet

A vitrified cell sheet recovered after rewarming, rehydration and removal of CPAs was transferred into Dulbecco’s phosphate-buffered saline (D-PBS; 10 ml) for washing. The cell sheet was cut into 1–2 mm^2^ pieces with ophthalmic scissors, and the pieces were incubated in RPMI-1640 medium containing 2 mg/ml collagenase II (17101; GIBCO) at 37°C for 40–50 min to isolate the cells. The suspension of isolated cells was centrifuged at 1,000 rpm for 5 min, and the precipitated cells were resuspended in RPMI-1640 medium (22400; GIBCO) and their viability was determined after trypan blue staining (viability (%) = live cells/live and dead cells × 100).

### Electron microscopy

Triple-layered chondrocyte sheets were soaked in 0.1 mol/l D-PBS containing 2% glutaraldehyde for 2 h, then fixed in 2% osmium tetroxide solution for 1 h, and then dehydrated through an ethanol series (50, 70, 80, 90, 95, and 100%). Next, the ethanol was replaced by 100% *tert*-butyl alcohol, and the samples were dried using freeze dryer (ES-2030; Hitachi High-Technologies Corporation, Tokyo, Japan). The dried specimens were sputter-coated with osmium and affixed to an adhesive interface for observation with a scanning electron microscope (JSM-6700 F; JEOL, Tokyo, Japan). The top surfaces of the cell sheets were observed at magnifications ranging from × 300 to × 2,000.

### Histological examination and immunohistochemical staining

Triple-layered cell sheets were harvested after culture or cryopreservation and fixed in 4% paraformaldehyde solution for 1 week. The specimens were embedded in paraffin and sectioned, and the sections were placed on glass slides. After deparaffinization and rehydration, the sections were stained for proteoglycan with 0.1% Safranin-O or immunostained with type II collagen antibody. For immunohistochemistry, the slides were incubated with a diluted primary anti-human type II collagen antibody (F-57: Daiichi Fine Chemical, Toyama, Japan) for 16 h at 4°C, followed by incubation with the EnVision + Mouse/HRP secondary antibody (K4000: DAKO, Glostrup, Denmark) for 1 h at room temperature. Finally, the sections were stained with diaminobenzidine (K3466: DAKO) and counterstained with hematoxylin. Coverslips were mounted onto the slides and sealed with nail polish. The slides were then examined under a microscope and images were captured (Biozero BZ-8000, KEYENCE, Osaka, Japan).

### Statistical analysis

Statistical analyses were performed using IBM SPSS Statistics 20.0 software (IBM Corporation, NY, USA). The proportional data were subjected to arcsine transformation and evaluated by one-way analysis of variance (ANOVA) followed by multiple comparisons using Tukey’s test. The level of significance was set at p values < 0.05.

## Results and discussion

### Maintenance of cell sheet structure and cell viability after vitrification

**Figure 3:**
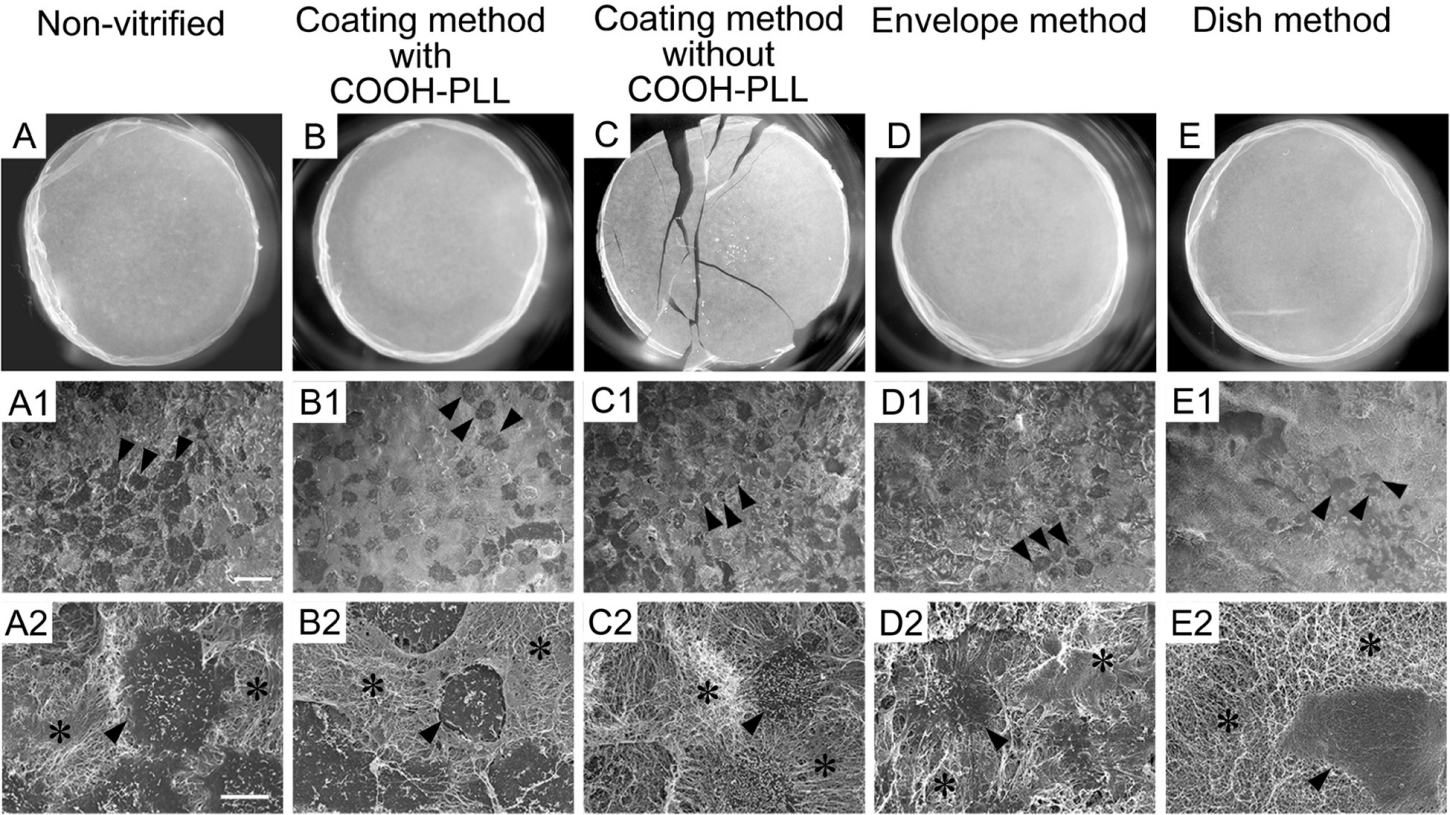
**The macro- and microstructures of triple-layered rabbit chondrocyte sheets after vitrification. A**-**E**: Morphological appearance of chondrocyte sheets after vitrification and rewarming. **A1**-**E2**: Scanning electron microscopic images of the surfaces of cell sheets recovered after vitrification and rewarming. **A1**-**E1**: ×300, Scale bar = 50 μm; **A2**-**E2**: ×2,000, Scale bar = 10 μm. The surfaces **(A1**-**E2)** are irregular, featuring pavement-like cell populations (arrowheads in **A1**-**E1** indicate representative three cells) and well-developed extracellular matrices with a dense fibrous structure (*). The microstructures of cell sheets vitrified by any of the methods described in this report were similar to those of the non-vitrified control sample.

Ten cell sheets were vitrified using the coating method in the presence of 10% COOH-PLL (Table 1). After rewarming, all the recovered sheets (100%) showed no visible damage and had same appearance as non-vitrified cell sheets (Figure 3A, B). Eight sheets were vitrified using the coating method in the absence of COOH-PLL (Table 1); only one of the recovered sheets (12.5%) did not have visible damage, all of the remainder exhibited cracks (Figure 3C). Cell viability in the vitrified cell sheets did not differ between protocols with or without COOH-PLL (92.1% vs. 91.9%); the rates of cell viability were comparable to that of the non-vitrified control (94.6%).

**Table 1:** Structural maintenance and cell viability after vitrification of triple-layered rabbit chondrocyte sheets

**Vitrification method**	Presence of COOH-PLL in VS*	**No. of cell sheets recovered without fracture / No. of cell sheets examined (%)**	**Cell viability (mean ± SEM)**
Non-vitrified control		8/8 (100)	94.6 ± 0.5^a^
Coating	+	10/10 (100)	92.1 ± 0.9^a^
	-	1/8 (12.5)	91.9 ± 0.7^a^
Envelope	+	7/7 (100)	86.8 ± 0.7^b^
Dish	+	7/7 (100)	77.6 ± 3.15^c^

^a-c^Values with different superscript differ significantly (p < 0.05).

*VS: Vitrification Solution.

These results clearly showed that vitrification by the coating method in the presence of COOH-PLL as a supplemental non-permeable CPA was capable of preserving the membranous structure of the cell sheet with a high survival rate for the constituent cells. To the best of our knowledge, our results represent the first successful vitrification of cell sheets grown in temperature-responsive dishes. In this study, we vitrified cell sheets in LN vapor rather than by direct immersion in the LN. This also had a critical influence on the maintenance of the membranous structure of the cell sheet during vitrification. In preliminary experiments, all of the cell sheets cracked, even in the presence of COOH-PLL, when they were vitrified by direct immersion in LN. Possibly, the direct immersion approach might have had a more drastic impact on membrane integrity than the vapor and, thereby, impaired cell sheet structure.

Since the coating method proved successful for vitrification of cell sheets, we examined whether cell sheets enveloped in a thin film could also be vitrified successfully. Seven cell sheets were placed into film envelopes, vitrified and rewarmed (Table 1). All the sheets (100%) were recovered without visible damage (Figure 3D). However, cell viability (86.8%) was slightly lower compared to that in the coating method (p < 0.05).

For use in clinical applications, it would be preferable if vitrified cell sheets could be stored and distributed in hygienic coverings. Our results demonstrated that a cell sheet enveloped in a thin film with a minimum volume of VS could be successfully vitrified. However, wrapping a cell sheet with a film might influence the optimal cooling and rewarming rates during vitrification and rewarming processes. As cooling and warming rates have a crucial influence on the viability of vitrified cells [34,35], it will be important to identify robust film materials that have high thermal conductivity and are protective against invasive pathogens, as well as improving cooling and rewarming methods.

We also examined the influence of the volume of vitrification solution on the morphology and survival of cell sheets. Seven cell sheets were vitrified using the dish method (Table 1) that involves a substantially greater volume of VS than the coating and envelope methods. VS containing COOH-PLL was used because a preliminary experiment revealed that its presence was essential to ensure crack-free vitrification using the dish method (Additional file 1: Figure S1). All of the 7 sheets (100%) that were vitrified were recovered without any cracks (Figure 3E). However, cell viability after vitrification was 77.6%, which was significantly lower than that observed following vitrification with the coating or envelope methods (p < 0.05).

High levels of viability can be achieved by minimizing the volume of the solution used for vitrification of mammalian embryos [31]. In contrast, the vitreous state becomes unstable when larger solution volumes are employed: more cracks tend to occur during the solidification of the solution and more ice crystals form upon rewarming. Our results demonstrated that addition of COOH-PLL as a non-permeable CPA was effective in stabilizing the vitrified state of the solution. However, even when the VS contained COOH-PLL, cell viability was reduced slightly with the dish method. The decrease might be attributed to the slower cooling and warming rates and/or ice crystal formation during rewarming. We observed that the VS appeared opaque for a moment during the rewarming process in the dish method, suggesting the occurrence of ice crystal formation.

### Scanning electron microscopic images of the surface of vitrified cell sheets

The microstructures of the cell sheets vitrified in the four experimental groups were compared to those of the non-vitrified sample (Figure 3). Although slight differences were observed among individual sheets, overall, the cell sheets retained their basic structure of pavement-like cells (Figure 3A1-E2) distributed within well-developed extracellular matrices (Figure 3A2-E2). The sheet surfaces were irregular, featuring well-developed extracellular matrices with dense fibrous structures (Figure 3A1-E2).

The microstructures of cell sheets were maintained in the vitrified samples of all the experimental groups under the same conditions as the non-vitrified control groups. The sheets that developed cracks during vitrification with the coating method in the absence of COOH-PLL showed no microstructural abnormalities (Figure 3C1, C2), suggesting that the fracturing of the sheet structure did not affect the microstructure. The sheets vitrified by the envelope method (Figure 3D1, D2) and the dish method (Figure 3E1, E2), where cell viabilities were slightly decreased, also exhibited no microstructural abnormalities. These results indicate that the microstructure of the vitrified cell sheet, including the extracellular matrix, were well maintained even after vitrification and rewarming under suboptimal conditions.

### Histological and immunohistochemical examination of vitrified cell sheets

**Figure 4:**
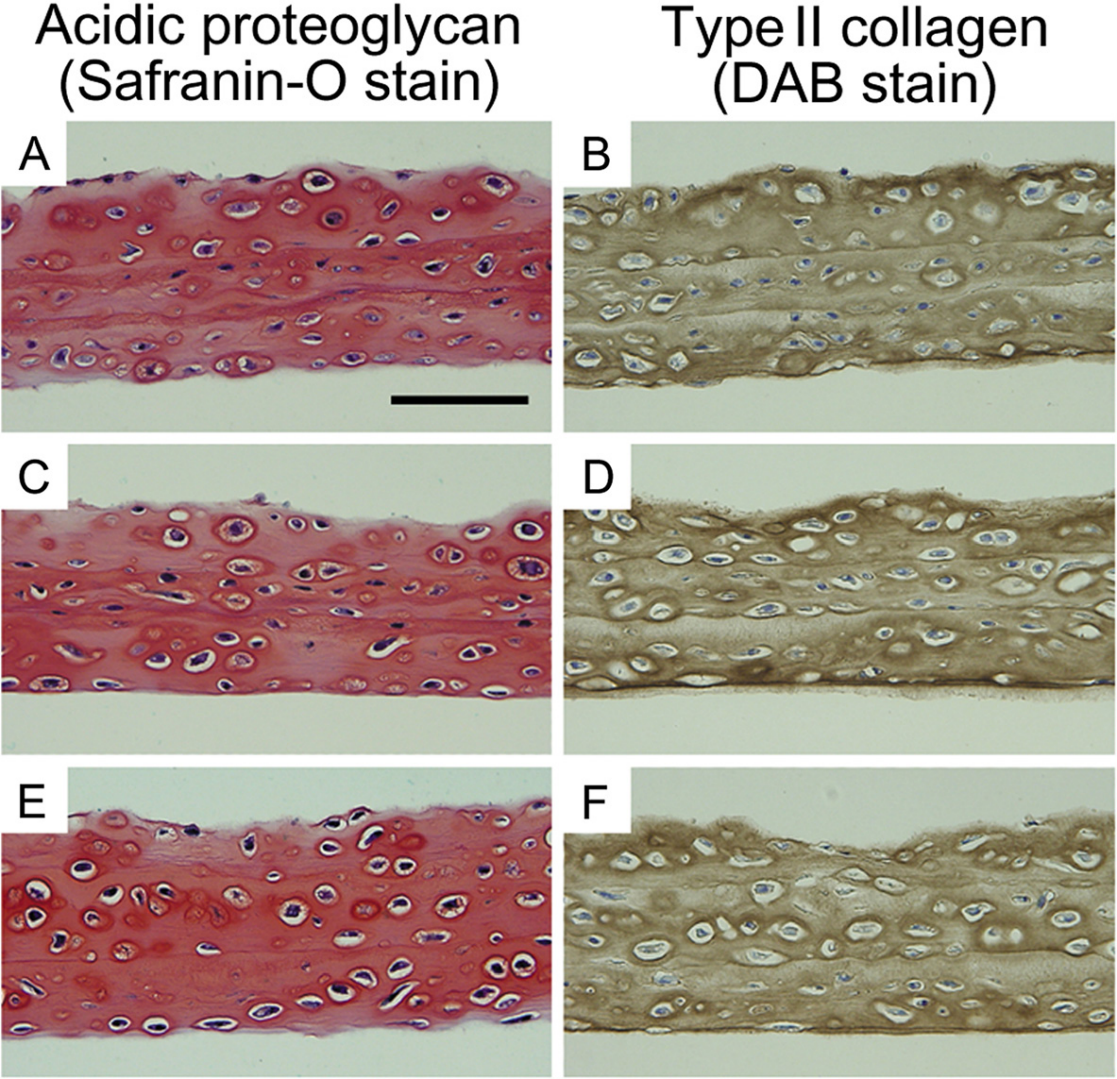
**Histological and immunohistochemical examination of triple-layered rabbit chondrocyte sheets.** Staining for proteoglycan **(A**, **C**, **E)** and type II collagen **(B**, **D**, **F)** on cross-sections of cell sheets recovered after vitrification and rewarming. **A**, **B**: Non-vitrified control cell sheet exhibiting large amounts of proteoglycan and type II collagen in the extracellular matrix. **C**, **D**: Cell sheet vitrified with the coating method using VS containing COOH-PLL; the sheet exhibits a normal extracellular matrix. **E**, **F**: Cell sheets vitrified by the envelope method. The extracellular matrix exhibits no difference to samples from control and coating method groups. (Scale bar = 100 μm).

Cell sheets vitrified with the coating and envelope methods in the presence of COOH-PLL were histochemically and immunohistochemically examined to investigate the distribution of the major components of cartilage, i.e. proteoglycan and type II collagen. In the non-vitrified control (Figure 4A) and the vitrified cell sheet, strong Safranin-O staining was exhibited (coating method: Figure 4C, envelope method: Figure 4E). These results showed that acidic proteoglycan were, in general, densely and evenly distributed throughout the chondrocyte sheet and this distribution pattern was maintained after vitrification in the coating and envelope methods.

The vitrified samples also exhibited large amounts of type II collagen (Figure 4D, F) in a similar manner as in the non-vitrified control (Figure 4B). Overall, these data showed that the extracellular matrix of the vitrified cell sheets had been maintained in both the coating and envelope methods.

### Significance of maintaining membranous structure in chondrocyte sheet cryopreservation

In the conventional slow-freezing method, cultured cell sheets are frozen in the presence of a relatively low concentration of a CPA [21]. Thus, extra- and intracellular ice crystal formation is inevitable during freezing, which destroys the cell sheet structure and decreases cell viability [21]. In contrast, with the vitrification method, a solution containing a high concentration of a CPA is rapidly cooled to achieve the transition from the liquid phase to the solid phase (amorphous) without ice crystal formation [36]. Therefore, cell sheets could be sealed in a glassy state that maintained their macro- and microstructures and also allowed high cell viability.

In cell sheet therapy, cytokines and growth factors produced by the cell sheet play an important role in healing damaged tissues [11,19,20]. We found that the formation of a chondrocyte sheet structure enhanced transforming growth factor-β secretion from the cells [37], which implies that maintaining the membranous structure after cryopreservation is a prerequisite for function. Therefore, in the present study, we focused on both the maintenance of sheet structures and of cell viability after vitrification. Thus, our study provides the first demonstration that cryopreservation of cultured chondrocyte sheets with a fragile membranous structure can be achieved using a vitrification method developed on the basis of the MVC concept. Biochemical functions such as cytokine production by the vitrified chondrocyte sheets has yet to be analyzed. Additionally, transplantation experiments using vitrified cell sheets are under consideration.

In our preliminary study, we could successfully vitrify cell sheets with more fragile characteristics including human chondrocyte sheets. It is, therefore, likely that the vitrification method developed in the present study can be applied to different types of cell sheet other than the triple layered rabbit chondrocyte sheets. In application of the vitrification technology to human therapies, toxicity of CPAs to human cells needs to be verified.

## Conclusions

In this study, we demonstrate that the vitrification method developed here facilitated the cryopreservation of a chondrocyte sheet while maintaining its macro- and microstructures and allowing a high rate of viability of the constituent cells. The coating method, where the cell sheet was vitrified with a minimum volume of VS in the presence of COOH-PLL, effectively prevented structural damage due to vitrification. Here, we propose three basic principles essential to the cryopreservation of chondrocyte sheets: (i) minimizing the volume of the vitrification solution by using the coating method, (ii) stabilizing the vitreous state via the addition of COOH-PLL as a non-permeable CPA, and (iii) preventing the occurrence of cracks in the vitrified solution by cooling samples in LN vapor instead of direct immersion into LN. The cryopreservation technology developed in this study will play a pivotal role in clinical applications of cell sheet-based therapies.

## Abbreviations

COOH-PLL: Carboxylated poly-L-lysine.

## Competing interests

The authors declare that they have no competing interests.

## Authors’ contributions

HN conceived and designed the experiments and wrote the manuscript. MM performed the experiments and wrote the manuscript together with HN. MW wrote the manuscript together with HN. TK and HM performed the experiments. MS (Michio Sato) scanned electron microscopic images. MY, MK prepared chondrocyte cell sheets. KM and HSH prepared COOH-PLL. MS (Masato Sato) and JM helped to draft the manuscript. All authors read and approved the final manuscript.

## Supplementary Material

Additional file 1: Figure S1 Protective effect of COOH-PLL against fracture of vitrified solution. COOH-PLL-free (A) and COOH-PLL-containing (B) solutions in the process of rewarming after vitrification in liquid nitrogen vapor. Note the occurrence of many cracks in the COOH-PLL-free solution (A), while the COOH-PLL-containing solution is free of cracks (B). The opacity of the solution in B indicates that ice crystals formed during the warming process. Click here for file
