# Supplementary material for: Development of a novel vitrification method for chondrocyte sheets
Source: BMC Biotechnol. 2013 Jul 25;13:58. doi: 10.1186/1472-6750-13-58 (PMC3726287; doi:10.1186/1472-6750-13-58)
Supplement: Additional file 1: Figure S1 — Protective effect of COOH-PLL against fracture of vitrified solution. COOH-PLL-free (A) and COOH-PLL-containing (B) solutions in the process of rewarming after vitrification in liquid nitrogen vapor. Note the occurrence of many cracks in the COOH-PLL-free solution (A), while the COOH-PLL-containing solution is free of cracks (B). The opacity of the solution in B indicates that ice crystals formed during the warming process. [file 1472-6750-13-58-S1.pdf]

Additional file

Additional file 1: Figure S1 Protective effect of COOH-PLL against fracture of vitrified solution.

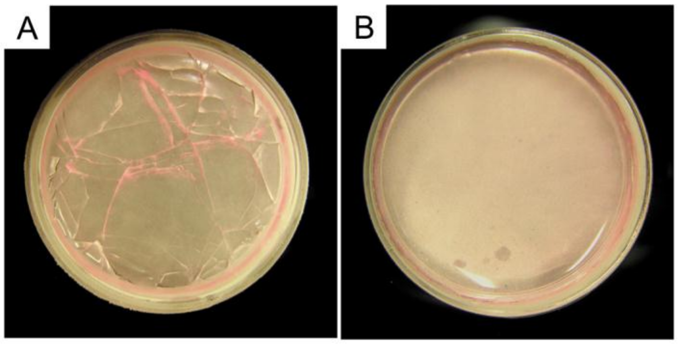

COOH-PLL-free (A) and COOH-PLL-containing (B) solutions in the process of rewarming after vitrification in liquid nitrogen vapor. Note the occurrence of many cracks in the COOH-PLL-free solution (A), while the COOH-PLL-containing solution is free of cracks (B). The opacity of the solution in B indicates that ice crystals formed during the warming process.
